# SARS-CoV-2 infection of intestinal epithelia cells sensed by RIG-I and DHX-15 evokes innate immune response and immune cross-talk

**DOI:** 10.3389/fcimb.2022.1035711

**Published:** 2023-02-07

**Authors:** Lijuan Zhang, Yize Zhang, Ruiqin Wang, Xiaoning Liu, Jinmeng Zhao, Masato Tsuda, You Li

**Affiliations:** ^1^ School of Medicine, Huanghe Science and Technology College, Zhengzhou, China; ^2^ Precision Medicine Center, Gene Hospital of Henan Province, The First Affiliated Hospital of Zhengzhou University, Zhengzhou, China; ^3^ School of Life Science and Technology, Tongji University, Shanghai, China; ^4^ School of Life Science, Zhengzhou University, Zhengzhou, China; ^5^ School of Medicine, Niigata University, Niigata, Japan

**Keywords:** SARS-CoV-2, IECs, RIG-I, DHX15, MAIT, IL-18

## Abstract

SARS-CoV-2 causes a spectrum of clinical symptoms from respiratory damage to gastrointestinal disorders. Intestinal infection of SARS-CoV-2 triggers immune response. However, the cellular mechanism that how SARS-CoV-2 initiates and induces intestinal immunity is not understood. Here, we exploited SARS-CoV-2-GFP/ΔN trVLP pseudo-virus system and demonstrated that RIG-I and DHX15 are required for sensing SARS-CoV-2 and inducing cellular immune response through MAVS signaling in intestinal epithelial cells (IECs) upon SARS-CoV-2 infection. NLRP6 also engages in the regulation of SARS-CoV-2 immunity by producing IL-18. Furthermore, primary cellular immune response provoked by SARS-CoV-2 in IECs further cascades activation of MAIT cells and produces cytotoxic cytokines including IFN-γ, granzyme B *via* an IL-18 dependent mechanism. These findings taken together unveil molecular basis of immune recognition in IECs in response to SARS-CoV-2, and provide insights that intestinal immune cross-talk with other immune cells triggers amplified immunity and probably contributes to immunopathogenesis of COVID-19.

## Introduction

The severe acute respiratory syndrome coronavirus 2 (SARS-CoV-2) pandemic has caused over 500 million infections and more than 6 million deaths worldwide (WHO, 2022). Infection with SARS-CoV-2 results in a spectrum of clinical presentations of varying severity. Besides the common respiratory symptoms, growing studies have reported a substantial proportion of gastrointestinal (GI) manifestations, such as nausea, abdominal pain, vomiting and diarrhoea at the onset of the disease (Xiao et al., 2020; Guo et al., 2021). Although the accurate mechanism of GI symptom is still unclear, intestinal organ-specific immune response has been proposed to link GI symptom in SARS-CoV-2-infected patient (Chen et al., 2022).

Clinical evidence shows that human intestine is susceptible to SARS-CoV-2 infection (Jin et al., 2020; Livanos et al., 2021). Intestinal enterocytes enrich ACE2 entry receptors and accessory proteinase TMPRSS2 which promotes SARS-CoV-2 virus efficiently infecting enterocytes in human intestinal organoids (Shang et al., 2020; Zang et al., 2020). Gastrointestinal tract provides the first line of host innate immune response for pathogenic microorganism invasion (Wu and Wu, 2012). *Ex vivo* studies of SARS-CoV-2 with human colon organoids or intestinal enteroids demonstrate that SARS-CoV-2 infection initiates antiviral innate immune response by producing type I interferon (IFN) and type III IFN (Stanifer et al., 2020; Zhou et al., 2020). While well-coordinated immune response induced by virus infection of epithelial cells leads to viral clearance, exuberant response and hyperactivation can contribute to disease exacerbation reversely. Patients infected with SARS-CoV-2 are also accompanied with aggressive inflammatory response and release of a large amount of pro-inflammatory cytokines (Divij et al., 2020; Laing et al., 2020; Leticia et al., 2020). In a nonhuman primate model, SARS-CoV-2 infection results in dynamic inflammation profile and inflammatory cytokines release including IL-1β, IL-1ra, MCP-1, IL-12, MIP-1β as disease progress in gastrointestinal tissue (Jiao et al., 2021). Of note, IL18 is upregulated at the onset of infection (dpi 1, 4), which is corresponding to that IL-18 is increased upon fever onset and remains highly elevated in the acute phase of SARS-CoV-2 infection in patients (Rodrigues et al., 2020a; Tao et al., 2020b; Flament et al., 2021; Ashrafzadeh-Kian et al., 2022). Thus, intestinal infection and replication of SARS-CoV-2 triggers antiviral or inflammatory response. Nonetheless, it remains unveiled that how SARS-CoV-2 infection initiates and induces immune response in intestine, and whether and how intestinal immunity participates in cross-talk in local immune response and even contributes to system cytokines production.

SARS-CoV-2 is a positive-sense, single-stranded RNA beta-coronavirus that produce double-stranded RNA (dsRNA) intermediates during genome replication and virion propagation (van Dorp et al., 2020; Wu et al., 2020). As in other coronavirus, dsRNA intermediates can be recognized by varied cytosolic RNA sensors, such as RIG-I, MDA5, DHX-15, LGP-2 which signals through MAVS and initiates antiviral innate immune response (Lu et al., 2014; Kell and Gale, 2015; Hur, 2019). Notably, the specific virus sensor that involves in different RNA viral defense is cell type dependent. In lung epithelial cells, MDA5 governs the innate immunity in response to SARS-CoV-2; while in macrophage, RIG-I dominates the immune response (Kato et al., 2006; Takeshi et al., 2007; Bruns et al., 2014; Kenta et al., 2014; Kell and Gale, 2015). Intestinal epithelia cells, the most abundant cell type in gut, are likewise equipped with various RNA sensors to defense viral invasion (Adrish et al., 2012; Lazear et al., 2015). It remains unclear whether SARS-CoV-2 immunity is subject to a similar sensor mechanism in intestinal epithelial cells and by which sensor virus are sensed to initiate immune response.

In viral infections, the crucial role of inflammasome in control of immune response and viral infections has been elucidated. NLRP3 inflammasome activation in infected macrophages/monocyte has been reported to contribute to SARS-CoV-2 related inflammation (Pan et al., 2021). A prominent role of Nlrp6 in the regulation of antiviral immune responses by interacting with dsRNA sensor DHX15 has also been documented recently (Xing et al., 2021). Nlrp6 exhibits cell-type specific higher expression in intestinal epithelial cells (Elinav et al., 2011) and mediates intestinal immunity. It is intriguing to investigate whether intestinal Nlrp6 inflammasome participates in SARS-CoV-2 related immune response in intestine.

Nlrp6 activation signals proinflammatory cytokine IL-18 production (Levy et al., 2015). In SARS-CoV-2 infected patients, IL-18 levels are markedly increased either in serum or faecal samples, which correlates with disease severity (Tao et al., 2020a). Furthermore, IL-18 mediated cell cross-talk in SARS-CoV-2 immunity subsequently cascades further immune response, and is associated with poor clinical outcome (Rodrigues et al., 2020a; Flament et al., 2021).

Here, we exploited SARS-CoV-2-GFP/ΔN trVLP system, as previously described (Ju et al., 2021), to infect intestinal epithelial cell line Caco-2 and investigated virus-enterocytes interaction to understand the mechanisms by which SARS-CoV-2 is recognized and induces immune response in intestinal epithelial cells. We found that RIG-I and DHX-15 are the required sensors for intestinal epithelial cells to recognize SARS-CoV-2 invasion and to induce IFNs and inflammatory cytokine production, particularly IL18. Coculture of SARS-CoV-2 infected intestinal epithelial cells with human PBMC results in activation of mucosal-associated invariant T cells and cytotoxic cytokine release such as IFN-γ, Granzyme B, TNF-α by MAIT in an IL-18 dependent manner. Given the elevated IL18 levels in relation to disease severity of COVID-19 patients, we proposed to link IL18 production to immunopathogenic response during SARS-CoV-2 infection.

## Result

### Entry receptor expression and permissiveness of intestinal epithelial cells to SARS-CoV-2 pseudo-virus

**Figure 1:**
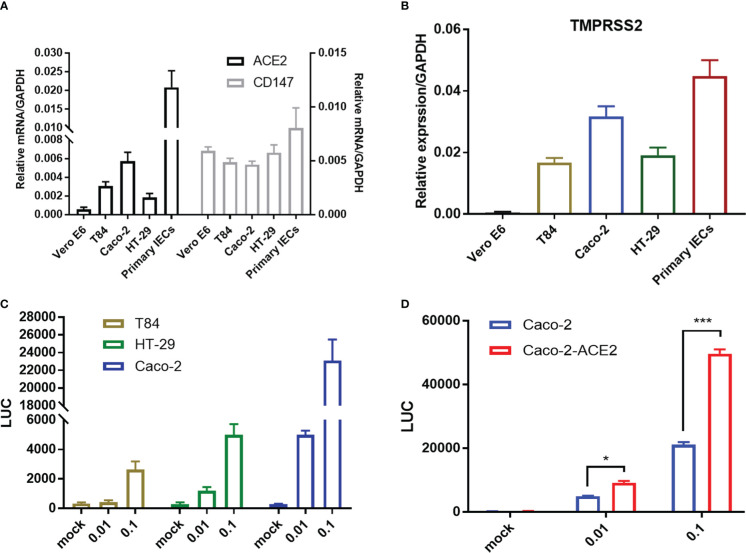
Entry receptor expression and permissiveness of intestinal epithelial cells to SARS-CoV-2 pseudo-virus. mRNA expression of SARS-CoV-2 entry receptor ACE2, CD147 **(A)** and coreceptor TMPRSS2 **(B)** on Vero E6, multiple epithelial cell lines and primary epithelial cells respectively. **(C)** Indicated intestinal epithelial cell lines (1x10^5^) were infected with VSV-SARS-CoV-2 pseudo-virus at increasing MOIs (0.01, 0.1). Luciferase activity was determined at 24 hpi. **(D)** Luciferase activity in ACE2 overexpressed Caco-2 cells (Caco-2-ACE2) at 24 hpi of VSV-SARS-CoV-2 pseudo-virus with increasing MOIs (0.01, 0.1). Data are represented as mean ± SEM. Statistical significance is indicated (*p<0.05, ***p<0.001, one-way ANOVA).

Intestinal epithelial cells (IECs) are highly permissive for SARS-CoV-2 infection due to high expression of entry receptor ACE2 and TMPRSS serine protease which facilitates virus entry into host cells (Zang et al., 2020). Recently, CD147-spike interaction was reported as an alternative entry route for SARS-CoV-2 infection (Wang et al., 2020). We screened related proteins expression in the frequently used intestinal epithelial cell lines, including T84, HT-29 and Caco-2, and primary IECs to seek an effective model for further investigation. Basically, all intestinal cell lines displayed constitutively high expression of ACE2, TMPRSS2, but not Vero E6. Of note, compared with T84 and HT-29, Caco-2 presented relative higher expression of ACE2 in mRNA and comparable CD147 (**Figures 1A, B**), corresponding to that Caco-2 is naturally permissive for SARS-CoV-2 infection and effective for viral replication (Stanifer et al., 2020). To determine the relative permissiveness of these intestinal cell lines to SARS-CoV-2 viral entry, we used a VSV-based SARS-CoV-2 pseudo-virus, designated as VSV-SARS-CoV-2, in which the backbone was provided by rVSV-ΔG-luciferase pseudotypes and the glycoprotein (G) gene was replaced with SARS-CoV-2 spike protein (Nie et al., 2020). Intestinal epithelial cell lines were inoculated with VSV-SARS-CoV-2 entry virus respectively and luciferase activity was analyzed at 24h post infection. The results largely correlated with ACE2 expression and Caco-2 presented highest luciferase activity after VSV-SARS-CoV-2 infection (**Figure 1C**), indicating the relatively higher permissiveness. Thus, we selected Caco-2 for further investigation. Given much higher ACE2 level in primary IECs, we enhanced ACE2 expression in Caco-2 by stable transduction, designated Caco-2-ACE2 (**Figure S1**). Permissiveness of Caco-2-ACE2 was measured with VSV-SARS-CoV-2 infection. Caco-2-ACE2 showed much higher luciferase activity than Caco-2 after 24h VSV-SARS-CoV-2 inoculation (**Figure 1D**).

### Establish pseudo-SARS-CoV-2 infection system in IECs mimicking viral life cycle

**Figure 2:**
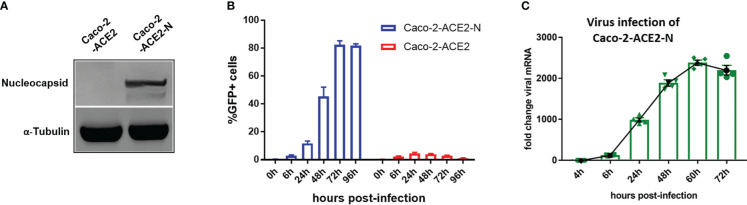
Establishment of pseudo-SARS-CoV-2 infection system in epithelial cell lines mimicking viral life cycle. **(A)** Caco-2-ACE2 cells were stably transfected with nucleocapsid. Western blot showed overexpression of nucleocapsid in Caco-2-ACE2-N cells. **(B)** Caco-2-ACE2 or Caco-2-ACE2-N cells (2x10^5^) were inoculated and infected with SARS-CoV-2 GFP/ΔN trVLP. GFP+ cell percentage was quantified by flow cytometry at indicated time points. **(C)** Replication kinetics of SARS-CoV-2 GFP/ΔN trVLP in Caco-2-ACE2-N cell was quantified with viral RNA expression by RT-PCR after inoculation.

Though VSV backbone-based SARS-CoV-2 pseudo-viruses have been broadly used to study SARS-CoV-2 entry and drug discovery (Hoffmann et al., 2020; Ou et al., 2020), these types of pseudo-virus are not applicable for simulating entire life cycle of SARS-CoV-2 since vesicular stomatitis virus (VSV) possesses divergent replication mechanism. Thus, we established an alternative pseudo-SARS-CoV-2 infection model in IECs to simulate viral life cycle, such as infection, replication and virion propagation, by exploiting transcription and replication-competent SARS-CoV-2 virus-like-particles (trVLP) system as previously described (Ju et al., 2021)with minor refinement. Briefly, reverse genetic viral genome fragments of SARS-CoV-2 were assembled as SARS-CoV-2-GFP/ΔN trVLP in which viral nucleocapsid gene (N domain) was replaced with GFP reporter; separately, viral N domain gene was stably assembled *via* lentiviral transduction into Caco-2-ACE2 cell (**Figure 2A**). The newly constructed cell line, designated as Caco-2-ACE2-N, and SARS-CoV-2-GFP/ΔN trVLP constituted the pseudo-SARS-CoV-2 infection model. Of note, the viral replication and complete life cycle of pseudo-SARS-CoV-2 was exclusively achieved in Caco-2-ACE2-N cells due to lacking of nucleocapsid gene in pseudo-SARS-CoV-2 genome. Thus, this system only required Biosafety level-2 environment. To validate the efficacy of infection and propagation of pseudo-SARS-CoV-2 system, *in vitro* transcribed SARS-CoV-2-GFP/ΔN mRNA was electroporated into Caco-2-ACE2-N cells to produce SARS-CoV-2-GFP/ΔN trVLP. The supernatant containing SARS-CoV-2-GFP/ΔN trVLP was collected and further inoculated Caco-2-ACE2 or Caco-2-ACE2-N cells respectively. SARS-CoV-2 GFP/ΔN trVLP infection developed increasing number of GFP+ Caco-2-ACE2-N cells over time, but barely GFP+ Caco-2-ACE2 cells were detected (**Figure 2B**). Concurrently, distinguishable replication kinetics were observed in Caco-2-ACE2-N cells and viral RNA multiplied and peaked at 60h dpi (**Figure 2C**). In contrast, GFP + cells or viral RNA was barely detectable or retain at low level in Caco-2 or Caco-2-ACE2 cells throughout the process (**Figures 2B, C**), which was in line with previously described. For sake of abbreviation, we used pseudo-SARS-CoV-2 or CoV-2 referring to SARS-CoV-2-GFP/ΔN trVLP below.

### Pseudo-SARS-CoV-2 infection induces innate immune response in intestinal epithelial cells

**Figure 3:**
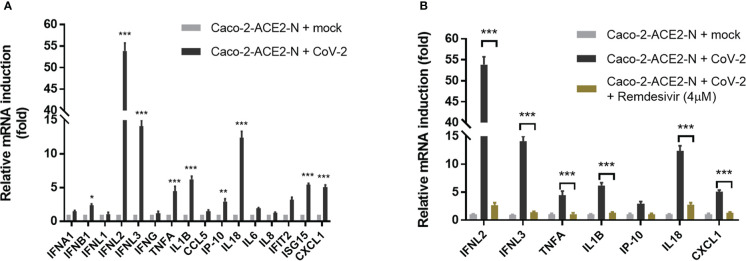
Immune response profile of Caco-2-ACE2-N cells to SARS-CoV-2 GFP/ΔN trVLP infection. **(A)** Caco-2-ACE2-N cells (2x10^5^) were infected with SARS-CoV-2 GFP/ΔN trVLP (CoV-2) with MOI = 0.25. At 48h post infection, mRNA induction of immune cytokines and chemokines response to CoV-2 infection was determined by qRT-PCR. Data were presented with fold change relative to mock infection. Four replicates were performed in the experiment. **(B)** With or without RNA replication inhibitor Remdesivir, the mRNA induction of immune cytokines and chemokines response to CoV-2 infection. Data are represented as mean ± SEM of four replicates. Statistical significance is indicated (*p<0.05, **p<0.01 ***p<0.001, unpaired t test).

Next, using the pseudo-SARS-CoV-2 infection system, we investigated the cellular immune response profile of Caco-2-ACE2-N cells to SARS-CoV-2 GFP/ΔN trVLP infection. Virus invasion basically initiates antiviral immune defense by producing interferons. We noticed that viral mRNA was declined over 72h post infection (**Figure 2C**). Thus, we measured the mRNA levels of IFNs over the course of infection by qRT-PCR first. As shown in **Figure S2A**, mRNA of IFN-λ2, IFN-λ3 was induced and peaked at 48-60h post infection, which was coordinated with time course of virus RNA levels. Two categories of immune factor genes containing anti-viral IFNs and inflammatory cytokines/chemokines commonly observed in COVID-19 patients were further determined. The mRNA expression of IFN-α, IFN-β, IFN-λ1, IFN-λ2/3, IFN-γ, TNF-α, IL-1β, CCL5, IP-10, IL-18, IL-6, IL-8, IFIT2, ISG15, CXCL1 was quantified at 48h post-infection. Generally, Caco-2-ACE2-N presented moderate immune response profile in response to pseudo-SARS-CoV-2 infection, which was in accordance with that reported in intestinal enteroids (Stanifer et al., 2020). Compared with non-infected cells, IFN-λ2, IFN-λ3, IL-18, IL-1β, ISG15 and CXCL1 were substantially induced by pseudo-SARS-CoV-2 infection in infected cells, while IFN-β, TNF-α, IP-10, IL-6, IFIT2 were moderately increased; other anti-viral cytokine IFN-α, IFN-λ1, as well as IFN-γ, CCL5, IP-10 were not induced significantly (**Figure 3A**). Furthermore, the immune response was aborted by Remdesivir which is incorporated into the viral RNA to inhibit RNA replication, suggesting that viral RNA replication may contribute to the induction of cellular immune response (**Figure 3B**). To further confirm the immune response induction at protein level, the elevated cytokines at protein level were measured by ELISA at 48h and 72h post-infection. IFN-λ2, IFN-λ3, IL-18, CXCL1 and TNF-α in Caco-2-ACE2-N cells was observed increased after exposure to pseudo-SARS-CoV-2 (**Figures S2B, C**). To address whether SARS-CoV-2 components themselves contribute to immune response, SARS-CoV-2-GFP/ΔN trVLP was irradiated by gamma ray and the incompetent virus were inoculated into Caco-2-ACE2-N cells for cytokine induction in IECs. The incompetent virus was not capable of inducing IFN-λ2, IFN-λ3, IL-18, CXCL1 and TNF-α production (**Figures S2B, C**). Collectively, these results indicated that pseudo-SARS-CoV-2 infection and propagation but not viral components themselves induced innate immune response in intestinal epithelial cells.

### RIG-I and DHX15 are required for cellular immune response in IECs upon SARS-CoV-2 infection

**Figure 4:**
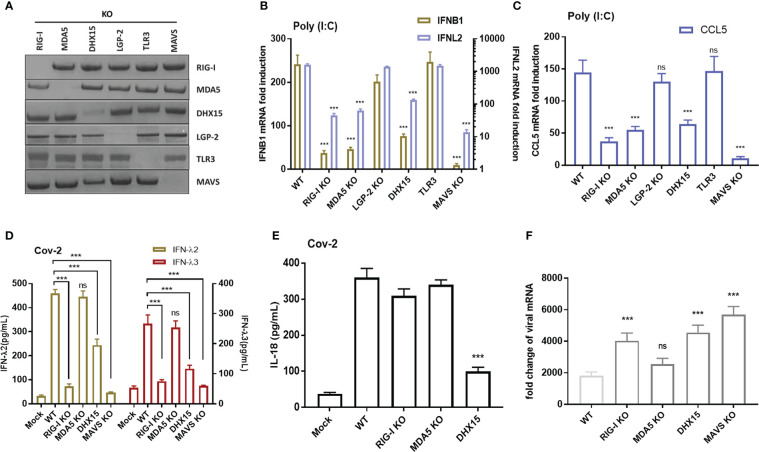
RIG-I and DHX15 are required for cellular immune response in IECs upon SARS-CoV-2 infection. **(A)** Western blot showing the knockout efficiency of multiple RNA sensors with CRISPR-Cas9 system in Caco-2-ACE2-N cells. The right indicated targeted immunoblot proteins. The top showed targeted knockout. **(B, C)** Cellular immune response to dsRNA analog poly I:C in RNA sensor knockout Caco-2-ACE2-N cells. The knockout cells were transfected with 5 μg/mL poly (I:C) to induce cellular immune response. After 16h post transfection, mRNA expression of IFNB1, IFNL2, CCL5 were quantified by qRT-PCR. Data are represented with fold changes relative to mock group. **(D, E)** Cellular immune response to CoV-2 infection in RNA sensor knockout Caco-2-ACE2-N cells . The knockout cells were infected with CoV-2 at 0.5 MOI. At 60h post infection, supernatants of cell infection culture were collected. IFNλ2, IFNλ3 and IL-18 were determined by ELISA in sensor knockout cells respectively. **(F)** Infected cells were also harvested; viral mRNA was quantified by qPCR. Fold change of viral RNA relative to mock group (no CoV-2 infection) was presented. Data are represented as mean ± SEM of four replicates. Statistical significance is indicated. (ns, not significant, ***p<0.001, unpaired t test and One-way ANOVA).

SARS-CoV-2 propagation produce double-stranded RNA (dsRNA) intermediates. Numerous viral RNA sensors have been reported to sense viral RNAs and initiate cellular immune response, of which RIG-I, MDA5, DHX-15, LGP-2 were demonstrated responsible for recognizing several enteric viruses, such as rotavirus, reovirus, EMCV. To identify the specific sensors that involve in recognizing SARS-CoV-2 RNA and contribute to immunity induction in intestinal epithelial cells, we established RNA sensors RIG-I, MDA5, DHX15, LGP-2, TLR3 deficient models with CRISPR-Cas9 knockout respectively in basis of Caco-2-ACE2-N cell line. In addition, the key adapter MAVS responsible for IFNs induction was also depleted efficiently for use (**Figure 4A**).

First, we exploit dsRNA analog poly I:C as a stimulus to investigate the immune response in RIG-I, MDA5, DHX-15, LGP -2, TLR3, MAVS depletion cell lines. Poly I:C stimulation of Caco-2-ACE2-N IEC engaged in immune response, presenting remarkably elevated IFNλ2, IFNβ, and CCL5. Depletion of RIG-I, MDA5 or DHX15 reduced the cytokine production with different extent in Caco-2-ACE2-N IECs, whereas depletion of LGP-2 or TLR3 had no effect on immune response (**Figures 4B, C**). These data implied critical role of RIG-I, MDA5 and DHX15 in recognizing RNA and regulating cellular immune response in Caco-2-ACE2-N IECs.

To investigate whether these findings are applicable in SARS-CoV-2-induced immune response, RIG-I, MDA5, DHX-15, LGP-2 or MAVS- knockout or control Caco-2-ACE2-N IECs were infected with SARS-CoV-2-GFP/ΔN-trVLP (MOI=0.5) and cytokine production was measured by ELISA. As shown in **Figures 4D, E**, depletion of RNA sensing adaptor RIG-I or DHX-15 diminished cytokine production in response to pseudo-SARS-CoV-2. IFN-λ2, IFN-λ3 expression were dramatically attenuated in RIG-I-KO Caco-2-ACE2-N IEC cells. DHX15-KO also impeded these cytokines production with slight but significant extent (**Figure 4D**). Notably, substantial IL-18 reduction was only observed in DHX15-KO Caco-2-ACE2-N IEC cells (**Figure 4E**). Depletion of MAVS, the key adaptor downstream of RIG-I or DHX-15, virtually abolished innate immune response to CoV-2 infection. In contrast, depletion of sensor MDA5 had little effect on cytokine production. These results indicated that RNA sensors RIG-I and DHX15 are required for cellular immune response to SARS-CoV-2 infection in Caco-2-ACE2-N IEC cells, and DHX15 is inferred to be responsible for IL-18 induction. Given the critical role of IFNs in viral inhibition, we investigated viral load in each group at 60h post inoculation. Viral replication and proliferation were derepressed in RIG-I or DHX15 -KO Caco-2-ACE2-N IECs, presenting higher level of viral mRNA in these cells compared to WT counterpart (**Figure 4F**).

### NLRP6 participated in the regulation of CoV-2 induced cellular immunity in IECs

**Figure 5:**
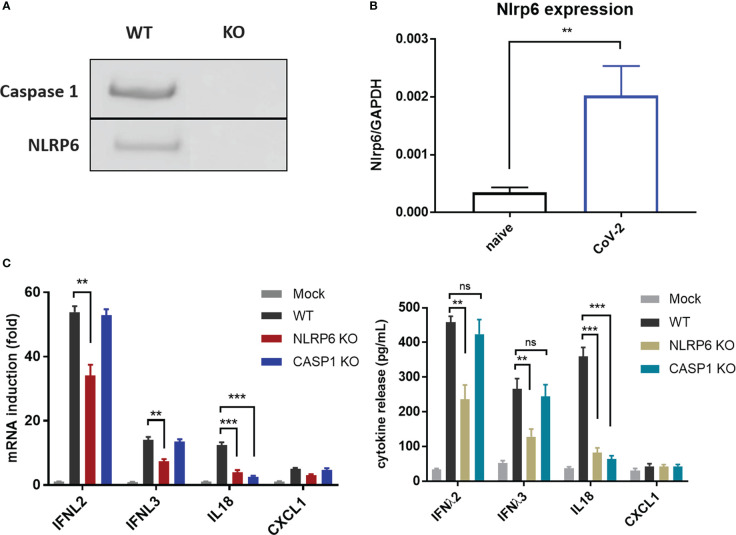
NLRP6 participated in the regulation of CoV-2 induced cellular immunity in IECs. **(A)** NLRP6 is constitutively expressed in Caco-2-ACE2-N cells. Western blot showing knockout efficiency of Caspase1 or NLRP6 by CRISPR-Cas9 system in Caco-2-ACE2-N cells. **(B)** Caco-2-ACE2-N cells were infected with CoV-2 at 0.5 MOI. At 16 hpi, mRNA level of nlrp6 was quantified by qRT-PCR. **(C)** The immune response profile of Nlrp6 or Casp1 knockout Caco-2-ACE2-N cells infected with CoV-2 for 48h. mRNA expression of anti-viral and inflammatory cytokines were determined by qPCR. Anti-viral and inflammatory cytokine release were confirmed at protein level by ELSIA. Data are represented as mean ± SEM of four replicates. Statistical significance is indicated. (ns, not significant, **p<0.01 ***p<0.001, unpaired t test).

Recently, NLRP6 was demonstrated to mediate cellular immunity in intestinal epithelia cells in response to enteric RNA virus infection, by interacting with DHX15. In addition, activation of NLRP6 inflammasome accounts for IL18 maturation and production. To determine whether NLRP6 engages in the regulation of SARS-CoV-2 immunity in IECs, we established NLRP6 knockout cell line in basis of Caco-2-ACE2-N IECs and evaluated the cytokine production with SARS-CoV-2 GFP/ΔN-trVLP infection. We confirmed constitutive expression of NLRP6 in Caco-2-ACE2-N IECs, and found SARS-CoV-2 infection. Incompetent SARS-CoV-2 virus were incapable of increasing NLRP6 expression (**Figure S3**) intriguingly upregulated NLRP6 expression (**Figures 5A, B**). In the context of SARS-CoV-2 GFP/ΔN-trVLP infection, cytokine production of IFNλ2/3, IL-18 (**Figure 5C**) was reduced in NLRP6-knockout Caco-2-ACE2-N IECs compared with parallel control either at mRNA or protein level, suggesting a pivotal role of NLRP6 in regulating SARS-CoV-2 immunity and induction of IFNλ2/3, IL-18 cytokine. To further define the role of caspase-1 which is downstream of NLRP6 inflammasome signaling, we evaluated cytokine production in Casp1-KO Caco-2-ACE2-N IECs. Only IL-18 production but not IFNs or CXCL1 were virtually disturbed in Casp1-KO Caco-2-ACE2-N IECs upon SARS-CoV-2 GFP/ΔN-trVLP infection, demonstrating that NLRP6-caspase1 axis is responsible for IL18 regulation in Caco-2-ACE2-N IECs (**Figure 5C**).

### Cross-talk between CoV-2-infected epithelial cells and immune cells provoked MAIT activation and exacerbated inflammation

**Figure 6:**
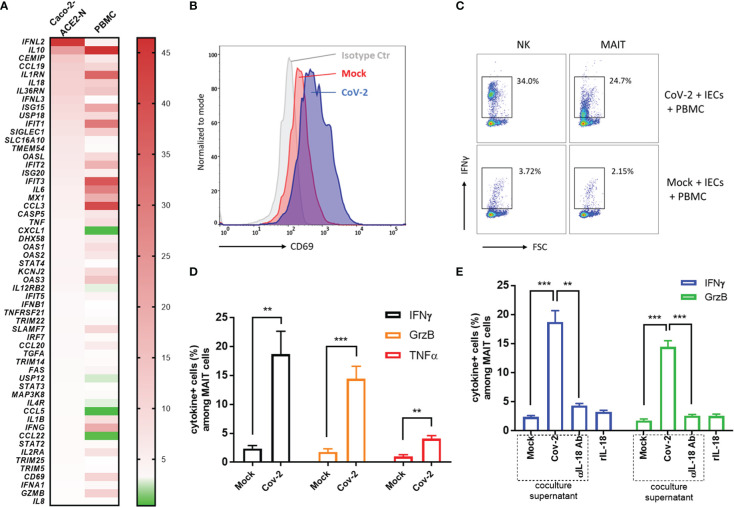
Cross-talk between CoV-2-infected epithelial cells and immune cells activated MAIT and exacerbated inflammation. **(A)** Heatmap of immunological profile of Caco-2-ACE2-N cells infected with CoV-2 and PBMC cocultured with CoV-2-infected Caco-2-ACE2-N cells. mRNA fold change of immune genes was profiled by RT2 Profiler PCR Array Gene Expression Analysis. Left panel indicated group of CoV-2-infected Caco-2-ACE2-N cells, shortly named with “Caco-2 ACE2-N”; data were presented with mRNA fold change relative to Mock-treated Caco-2-ACE2-N cells. Right panel indicated group of PBMCs cocultured with CoV-2-infected Caco-2-ACE2-N cells, shortly named with “PBMC”; data were presented with mRNA fold change relative to control PBMCs cocultured with Mock-treated Caco-2-ACE2-N cells. **(B)** CD69 expression profile on MAIT cells in various coculture groups. “Isotype ctr” indicated isotype antibody staining; “Mock” group indicated MAIT cells cocultured with Mock-treated IECs (Caco-2-ACE-2-N); “CoV-2” group indicated MAIT cells cocultured with CoV-2-infected IECs. **(C)** Representative flow cytometry dot plot of IFNγ expression in NK and MAIT cells after coculture with either Mock or CoV-2 infected IECs (Caco-2-ACE-2-N). **(D)** Inflammatory cytokine (IFNγ, GrzB, TNFα) expression in MAIT cells determined by FACS with positive population percentage. “Mock” indicated group of MAIT cells cocultured with Mock-treated IECs; “CoV-2” indicated group of MAIT cells cocultured with CoV-2-infected IECs **(E)** Depletion of IL18 by adding αIL18 Ab into medium at the beginning of coculture. Cytokine expression in MAIT with depletion of IL18 in coculture or with rIL18 addition alone in PBMC culture medium. All data are represented as mean ± SEM of three replicates. Statistical significance is indicated. (ns, not significant, **p<0.01 ***p<0.001, unpaired t test or One-way ANOVA).

The host’s immune system plays a pivotal role in determination of the course of disease of COVID-19. Primary immune response is required for pathogen elimination during onset stage, while overbalanced immune response is deleterious and contributes to systemic hyperinflammation and local damage. To better understand immune dysregulation and immunopathogenesis in COVID-19, we established an *in vitro* coculture system in which Caco-2-ACE2-N IECs were exposed to SARS-CoV-2 GFP/ΔN-trVLP for 48h; supernatants were replaced with fresh medium suitable for PBMCs; the infected IECs were then cocultured for 16h with human peripheral blood mononuclear cells (PBMCs) in Transwells. Transcriptional change of immunity related genes was profiled with RT2 Profiler PCR Array Gene Expression Analysis. We found that CoV-2 epithelia-immunocytes coculture induced a profound inflammatory response in immunocytes, distinct from the immunological profile of Caco-2-ACE2-N cells infected with SARS-CoV-2 (**Figure 6A**). Of note, in PBMC immunocytes, anti-viral genes or interferon induced genes, such as ISG15, IFIT1, IFIT2, IFIT3, MX1, OAS3, OAS1, were substantially elevated by the coculture. Inflammatory cytokines, such as IL10, IL6, CCL3, IL1B, IFNG, GZMB or receptors, IL2RA, IL1RN were as well activated in PBMC by coculture. These data suggested that cellular cross-talk contributed to further immune response and magnification. Recent study reported mucosal-associated invariant T (MAIT) cells in particularly are highly activated in COVID-19 patients, and corelated with cytotoxicity by producing IFN-γ, Granzyme B, TNF-α cytokines, and with the severity and mortality of SARS-CoV-2 (Flament et al., 2021). Proinflammatory cytokines, notably interleukin (IL)-18, was associated with MAIT activation. Given the fact that IL-18 receptor is highly expressed on MAIT cells, and high level of IL-18 was induced in intestinal epithelia cell in response to SARS-CoV-2 infection, we sought to examine whether SARS-CoV-2 infection of IECs contributes to MAIT activation and potentiate further inflammatory response. Flow cytometry analysis showed that after coculture with CoV-2 infected Caco-2-ACE2-N cells, CD69 was upregulated in CD161+Vα7.2+ MAIT cells, but not in other T cell subtypes (**Figure 6B**). CoV-2 infection of IECs stimulated robust IFN-γ, Granzyme B, TNF-α production in MAIT cells (**Figures 6C, D**). Additionally, substantial IFN-γ induction was also observed in NK cells (**Figure 6C**). Exposure to the conditional medium from infected IECs presented a similar activation profile in MAIT, suggesting that some factors in conditional medium activated PBMCs and MAIT. We speculated that IL-18, originally named IFN-γ inducing factor, contributed to MAIT activation and IFN-γ production. Indeed, depletion of IL-18 from conditional medium with αIL-18 failed to induce IFN-γ production in MAIT cells. However, recombinant IL-18 alone was incapable of activating MAIT cells, suggesting that IL-18 is required but not sufficient for MAIT activation (**Figure 6E**). Taken together, these data indicated that SARS-CoV-2 infection initiates primary cellular immune response in IECs, which cascades further activation of PBMCs and MAIT cells to produce inflammatory cytokines including IFN-γ, granzyme B *via* an IL-18 dependent mechanism.

## Discussion

Infection with SARS-CoV-2 causes a spectrum of clinical symptoms of varying severity from respiratory damage to gastrointestinal disorders. Although studies have evidenced that human intestine is highly susceptible to SARS-CoV-2 infection, the mechanism how SARS-CoV-2 is sensed by intestinal cell host and induces cellular response is unclear. Innate immune response is pivotal for viral elimination at onset stage by producing IFN and proinflammatory cytokines. However, overbalanced immune response instead contributes to pathogenesis of COVID-19, such as acute lung injury, GI symptoms and even systemic inflammatory response syndrome (SIRS) (Polidoro et al., 2020; Sherwani and Khan, 2020). Gastrointestinal system, as an immune organ, have been proposed to play a crucial role in SARS-CoV-2 immunity. Therefore, it is urgent and indispensable to investigate the signaling pathways and mechanisms that contribute to cellular immune response in intestinal cells upon SARS-CoV-2 infection.

Manipulating infectious pathogenic virus is strictly restricted in BSL-3 laboratories, which hinders broad experimental research. Reverse genetics have profoundly advanced experimental viral study. Recently, several reverse genetics systems have been successfully created to produce fully infectious recombinant SARS-CoV-2 virus and replicons, providing powerful tools for broader study on SARS-CoV-2 beyond clinical isolates (Shang et al., 2020; Zang et al., 2020). In this concept, Ju et al. (2021) developed a trans-complementary system modeling SARS-CoV-2 life cycle (Ju et al., 2021), which only required biosafety 2 level environment. We exploited the trans-complementary system to mimic SARS-CoV-2 infection in intestinal cells and investigated the underlying mechanism of immunity.

In this study, we found that unlike lung epithelial cells, intestinal epithelial cell line Caco-2 presented relatively moderate immune response upon SARS-CoV-2 infection. Specifically, canonical anti-virus IFN, IFN-β, was not significantly induced, whereas IFNλ was dominant in anti-virus cellular immune response, which is coordinated with the findings in colon enteroid studies in response to SARS-CoV-2. The moderate anti-viral IFN production may partially explain why human colon epithelial cell line Caco-2 can support extensive viral replication and produce large amounts of viral particles in infection. ISG gene activation indicated inflammatory immune response. Of the induced proinflammatory cytokines, IL-18 was substantially increased. TNF-α, CXCL1, IP-10 were also mounted in the context of SARS-CoV-2 infection at variant timepoints. Thus, SARS-CoV-2 infection induces moderate but considerable anti-virus and inflammatory immune response in intestinal epithelial cells. The recently epidemic Omicron variant displayed less pathogenic in clinical. Research study reported that unlike previous SARS-COV-2 variant, Omicron isolates present less replication and lower viral load in Caco-2 cell when infected, and are highly sensitive to interferon treatment (Bojkova et al., 2022). This suggested sufficient IFNs were produced by intestinal epithelial cells to control virus, in agreement with less GI symptoms and less severe disease in omicron infected patients.

SARS-CoV-2 is a positive-sense RNA virus belonging to the β-coronavirus genus. The innate immune system defense against RNA virus infection by developing multiple RNA recognizing mechanisms to induce numerous host defense molecules, including type I and type III interferons and proinflammatory cytokines and chemokines. The RLRs are commonly identified sensors that recognize viral RNA in the cytosol and are essential for triggering the innate immune response to RNA viruses in most cell types (Loo and Gale, 2011; Chow et al., 2018). The RLRs include MDA5, LGP2 and RIG-I. Recently, MDA5 and RIG-I was shown to sense SARS-CoV-2 and trigger IFN production in lung epithelial cells (Yin et al., 2021; Antoine et al., 2022). Several members of the DExD/H-box helicases other than the RLRs have emerged as important for innate immune signaling and control of virus infection. DHX15 has been characterized as a sensor for several enteric RNA viruses and control production of IFN-β, IFN-λ3, and IL-18 in IECs (Kenta et al., 2014; Lu et al., 2014; Xing et al., 2021). Recent study identified DHX16 recognizes specific viral RNA to trigger RIG-I-dependent IFN-β production (Hage et al., 2022).

We also demonstrated that RIG-I and DHX15 were required for sensing SARS-CoV-2 and inducing cellular immune response through MAVS signaling in intestinal epithelial cells upon SARS-CoV-2 infection. Of note, RIG-I was mainly responsible for antiviral immunity and inflammatory cytokine production, while DHX15 also participated in regulation of IFNs but was specifically responsible for IL-18 production. The overlapping but distinctive contribution to SARS-CoV-2 immunity suggested interaction between DHX15 and RIG-I signaling. DHX15 appeared to interact with both K63- and K48-poly-Ub (Hage et al., 2022). Unanchored K63-linked poly-Ub (K63-poly-Ub) promotes the activation of RIG-I for optimal production of IFNs and pro-inflammatory cytokines (Zeng et al., 2010; Jiang et al., 2012). These clues hint that DHX15 may interact with K63-poly-Ub and partner with RIG-I for antiviral IFNs production and ISG expression. Depletion of MAVS shut down the immune response, implying a vital role of MAVS in the signaling.

Furthermore, we found that NLRP6 engaged in the regulation of SARS-CoV-2 immunity in IECs. Depletion of NLRP6 diminished the immune response and cytokine production, especially IL-18. It has been reported that DHX15–NLRP6 sense RNA and subsequently regulate IFNs and IL-18 secretion in the control of intestinal viral infections (Xing et al., 2021). However, another study showed that loss of DHX15 has no impact on inflammasome activation during virus infection, suggesting a DHX15-independent manner of NLRP6 inflammasome activation (Shen et al., 2021). Thus, it remains unclear whether DHX15 links to NLRP6 and how NLRP6 achieves roles in SARS-CoV-2 immunity in IECs. Notably, recombinant IFN-L2 treatment induces Nlrp6 mRNA expression in IECs (Penghua et al., 2015). It is possible that RIG-I- and DHX15-MAVS signaling produces type III IFNs, which further induces Nlrp6 expression and results in Nlrp6 inflammasome activation.

Infection of SARS-CoV-2 potentially provokes excessive production of proinflammatory cytokines and even systemic hyperinflammation, resulting in multiorgan failure to lethal damage. A few research studies have emphasized that immune dysregulation contributes to SARS-CoV-2 immunopathogenesis, dissecting the molecular mechanism that profound alteration in myeloid cells, T cells drives hyperinflammation and severe clinical manifestations (le Bert et al., 2020; Lu et al., 2021). In our study, SARS-CoV-2 infection of intestinal epithelial cells is characterized with excessive IL-18 production. Using coculture system, we investigated the interaction between SARS-CoV-2 infected intestinal epithelial cell Caco-2 and PBMCs. SARS-CoV-2 infected IECs directly produced mixed antiviral and inflammatory cytokines. MAIT cells in PBMCs was activated when cocultured with infected IECs in an IL-18-dependent mode and subsequently amplified the immune response by producing IFN-γ, TNF-α and granzyme B. As data showed, blocking IL-18 in SARS-CoV-2 infected IECs conditional medium with IL-18 antibody interrupted MAIT activation, while recombinant IL-18 alone could not activate MAIT, suggesting that IL-18 is required for MAIT activation but not sufficient and other supernatant factors or cell interactions are required in IECs- immunocytes cross-talk for MAIT activation. Granzyme B was reported to activate cell pyroptosis (Verdonck et al., 2022). Recent study demonstrated that IFN-γ and TNF-α combination triggers Inflammatory Cell Death, Tissue Damage, and Mortality in SARS-CoV-2 Infection and Cytokine Shock Syndromes (Karki et al., 2021). Given the prominence of MAIT cells in human peripheral blood (1–10%) and in tissue sites of inflammation (Dusseaux et al., 2011; Fergusson et al., 2016), MAIT activation may considerably account for systemic hyperinflammation and local damage in SARS-CoV-2 infection. Our data provides insights that cell cross-talk in SARS-CoV-2 infection triggers cascade and amplified immunity and contributes to immunopathogenesis of COVID-19.

**Figure 7:**
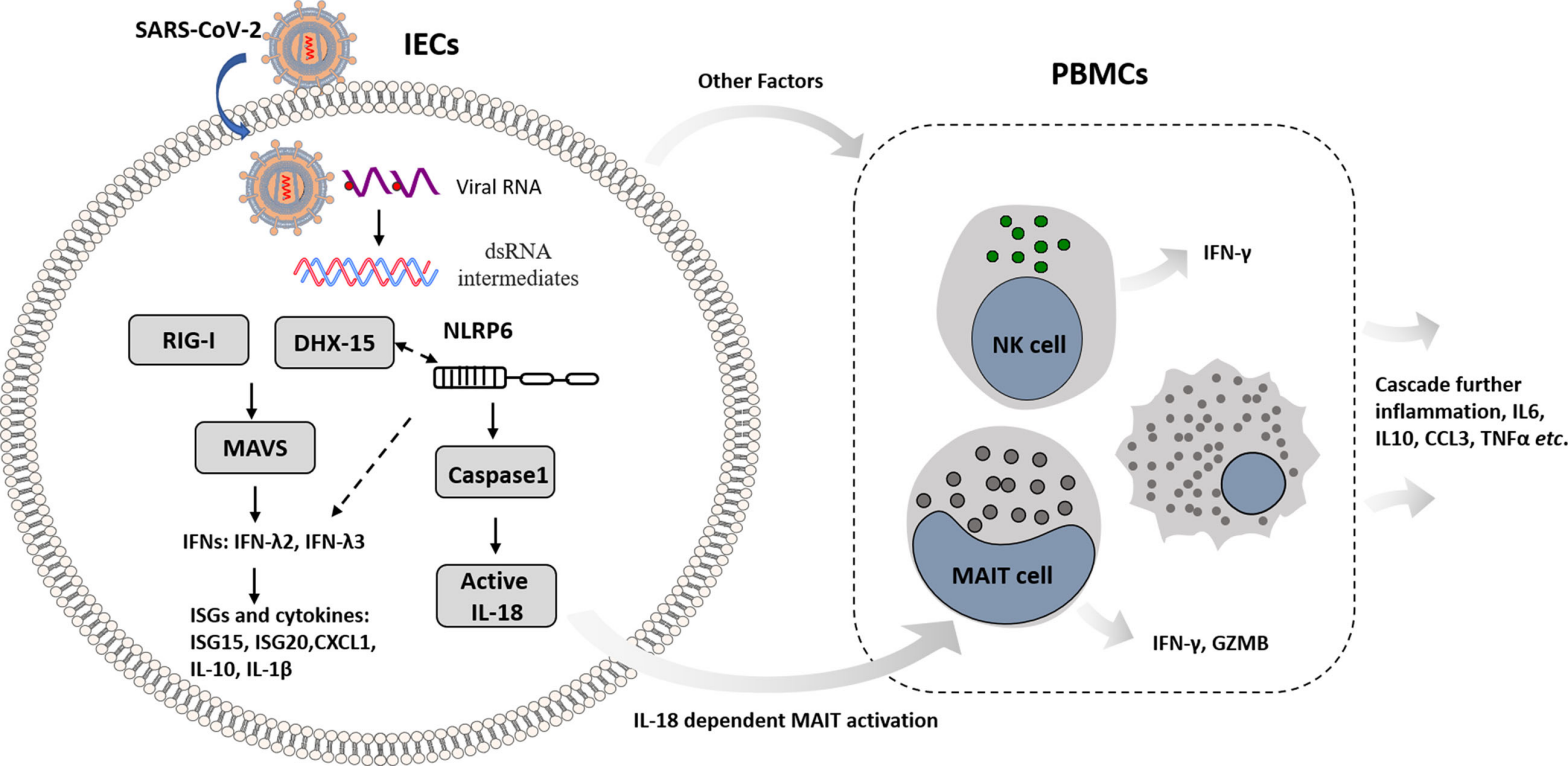
Schematic overview of mechanism basis of innate immunity in IECs response to SARS-CoV-2 infection and immune cross-talk with other immune cells in intestinal context.

In summary, this work unveils the molecular basis of immune recognition of the SARS-CoV-2 virus, depicts mechanism of cellular response in IECs, and provides insights that intestinal immune cross-talk with other immune cells triggers amplified immunity (**Figure 7**). Nevertheless, the viral immunity driven by innate and adaptive immune response balances host immunopathogenesis and protective effect with great complexity. DHX-15-MAVS signaling pathway cross-talks with NLRP6 related inflammasome in IECs; IECs transmit immune response to mucosal and peripheral immune cells, resulting in overall response. We propose further studies to dissect the specific mechanism that links viral sensor DHX-15 with NLRP6 signaling pathway. Also, it is critical to fully understand the immune cross-talk between distinct immune cell types, such as MAIT, NK, monocytes, and T cells, in which various cytokines besides IL-18 involved. These insights would benefit COVID-19 prevention and therapeutics development.

## Method

### Cell culture

Human colon carcinoma cell line T84 (ATCC CCL-248) and human intestinal epithelial cells (IECs) line HT-29 (ATCC HTB-38) were maintained in DMEM/F12 medium supplemented with 10% fetal bovine serum (FBS) and 1% penicillin/streptomycin. Human colorectal adenocarcinoma Caco-2 (ATCC HTB-37) and Vero E6 (ATCC CRL 1586) were maintained in DMEM supplemented with 10% FBS and 1% penicillin/streptomycin.

Mouse primary intestinal epithelial cells (IECs) were isolated from 6-week-old C57BL/6 mouse intestines. Briefly, intestines were cut into pieces and digested with 0.72mg/mL Dispase and 2.5mg/mL Collagenase IV for 30min. Cell suspension was collected and separated by Percoll gradient (GE Health). Purified IECs were cultured in DMEM, supplemented with 10% FBS, 4mM glutamine, 20mM HEPES, and 1% penicillin/streptomycin. After culture, the purity of IECs was confirmed by flow cytometry with PE-anti-E-Cadherin antibody and FITC-anti-Cytokeratin 18 antibody.

Caco-2 cells stably expressing ACE2 and nucleocapsid were generated by transduction with lentivirus containing ACE2/IRES-puro or nucleocapsid/IRES-G418 vector. Stably transduced cells were maintained in corresponding culture medium with 1μg/ml puromycin, and/or 1 mg/ml G418.

For CRISPR-Cas9-mediated gene disruption, Caco-2-ACE2-N cells were transduced with Cas9 and sgRNA LentiVector and selected with antibiotics for at least 10 days. cells were subcloned and then lysed for western blot to identify knockout clones.

### Pseudo-SARS-CoV-2 entry virus infections

To determine permissiveness of intestinal cell lines to SARS-CoV-2 viral entry, pseudo-typed virus VSV-SARS-CoV-2 with luciferase was used for infection assay. Cells were seeded in 96 well plates. VSV-SARS-CoV-2 was added at the indicated MOI. At 24 hpi, cells were harvested for luciferase assays following the Luciferase Assay System protocol (E1501, Promega). Luminescence was detected by Perkinelmer EnSight Microplate Reader.

### Virus titration assay

Viral titers in cell culture supernatant were determined by a modified plaque-forming assay with Caco-ACE2-N cells. Briefly, 5x10^5^ Caco-ACE2-N cells were seeded in 12-well plates overnight. Harvested culture supernatants from infected Caco-ACE2-N cells were 10-fold serially diluted with fresh medium. Seeded cells were inoculated with diluted supernatants respectively. After 1h infection, 1 mL of 0.6% microcrystalline cellulose (MCC, Sigma 435244) was added. The plates were incubated for another 3 days in incubator. On day 4, MCC was removed. 4% formalin was used to fix the cells. Plaques were quantified and recorded as PFU/mL.

### Quantitative reverse-transcription-PCR

Total cellular RNA was isolated using Qiagen RNeasy Mini Kit according to the manufacturer’s instructions. Reverse transcription was performed using iScript™ cDNA Synthesis Kit (Bio-Rad). The primers for viral RNA were as follows: Vprimer1-CGAAAGGTAAGATGGAGAGCC and Vprimer2- TGTTGACGTGCCTCTGATAAG. Real-time qPCR was performed in 96-well plates in triplicates using SYBR Green Supermix (Bio-Rad) and StepOne Plus system (Applied Biosystems). For other immune genes detection, Taqman probes, GAPDH (Hs99999905_m1), ISG15 (Hs01921425_s1), OAS1 (Hs00973637_m1), IFITM3 (Hs03057129_s1), IFNB1 (Hs01077958_s1), IFNL1 (Hs00601677_g1), and IFNL2 (Hs00820125_g1), etc. were used for quantification of mRNA expression.

### RT2 profiler

The RT2 first strand kit (Qiagen) was used for the synthesis of the cDNA strand using 500ng of total RNA from samples extracted using the RNeasy kit (Qiagen). The customized RT2 Profiler PCR array kit was used for profiling immunological genes expression according to the manufacturer’s instructions. The average of the threshold cycle (CT) values from mock controls were used to normalize gene expression. Fold changes of mRNA expression between the noninfected and the infected conditions were analyzed using the ΔΔCT values.

### ELISA

The supernatant from different groups were collected and cytokines were measured by human IFN-λ2, IFN-λ3, IL-18, CXCL1 and TNF-α ELISA kit (Biolegend) following manufacturer’s instructions.

### Coculture of CoV-2 infected IECs with PBMC

Human PBMC were purchased from SAILY Bio. 1x10^6^ PBMCs were cocultured with 2×10^5^ SARS-CoV-2 GFP/ΔN trVLP-infected (MOI=0.5) or mock Caco-2-ACE2-N cells in a 96-well U-bottomed plate and incubated for 16 h with the addition of brefeldin A at 10 h. After 16h coculture, cells were harvested and fixed with Fixation Buffer (Biolegend) for FACS staining.

For Transwell coculture, Caco-2-ACE2-N cells were inoculated and infected with mock control or pseuo-SARS-CoV-2. After 48h inoculation, supernatants were replaced with fresh medium suitable for PBMC. Then CoV-2-infected or mock Caco-2-ACE2-N cells were replated onto Transwell inserts. PBMCs were placed into the bottom chamber for 16h of coculture. After that, PBMC were harvested for immune genes profiling with customized RT2 Profiler PCR Array Gene Expression Kit (Qiagen). Cocultures were performed with at least three biological replicates per condition. For depletion assay, 20μg/mL αIL-18 neutralizing antibody was added at the start of the coculture. For conditional medium assay, conditional medium of Caco-2-ACE2-N cells with 60h CoV-2 infection was added into PBMC with 1:1 ratio of PBMC culture medium. After 16h culturing, PBMC were harvested and MAIT cells were analyzed by flow cytometry. For rIL18 addition, 10ng/mL rIL18 was added into PBMC culture for 16hs.

### Flow cytometry

Flow cytometry was exploited for analysis of cytokine expression after coculture. Surface staining was performed as follows. MAIT cells were defined by anti-CD3-FITC (BioLegend), anti-CD161-APC/Cy7 (BD Pharmingen) and anti-Vα7.2-APC (BD Pharmingen). NK cells were identified by anti-CD56-BV421 (BioLegend). After surface staining, cells were aliquoted into 3 parts and incubated with anti-IFNγ-PE (clone B27), anti-TNFα-PE (clone MAb11), or anti-GzB-PE (clone GB11) for intracellular staining at 4°C for 30min. Dead cells were excluded with 7-AAD. Cells were acquired on BD Lyric analyzer (BD Biosciences) and analyzed with FlowJo software.

### Western blot

Cells were washed twice with PBS on ice and lysed with lysis buffer (50 mM Tris-HCl, 1 mM EDTA, 150 mM NaCl, 1.0% NP-40) containing protease inhibitor Cocktail (ThermoFisher Scientific) for immunoblot analysis. Protein samples were dissolved in SDS sample buffer. After electrophoresis, separated proteins were transferred onto PVDF membrane. The membrane was then blocked with 5% nonfat milk. After incubation with specific primary antibody, HRP-conjugated secondary antibody was applied. The membranes were scanned by an enhanced chemiluminescence system (ThermoFisher Scientific).

### Quantification and statistical analysis

All data were presented as means ± SEM as indicated and analyzed by GraphPad PRISM software. Unpaired t test, Student’s paired t test, one-way ANOVA with multiple comparisons were used. *p < 0.05; **p < 0.01; ***p < 0.001.

## Data availability statement

The original contributions presented in the study are included in the article/**Supplementary Material**. Further inquiries can be directed to the corresponding authors.

## Author contributions

YL designed the experiments and wrote the manuscript. LZ, YZ and RW mainly designed and conducted the experiment. LZ and YZ contributed to equal work. RW conducted flow cytometry, qPCR and cell culture. XL completed molecular construction and provided plasmids. JZ performed western blot and ELISA, MT helped to revise the manuscript. All authors contributed to the article and approved the submitted version.

## References

[B1] Adrish S. Michael R. Gourab M. Ningguo F. Tomer K. Nitya N.. (2012). Innate immune response to homologous rotavirus infection in the small intestinal villous epithelium at single-cell resolution. Proc. Natl. Acad. Sci. 109, 20667–20672. doi: 10.1073/pnas.1212188109 23188796 PMC3528539

[B2] Antoine R. Luiza C. V. A. Marine T. Ghizlane M. Boris B. Joe M.. (2022). SARS-CoV-2 triggers an MDA-5-Dependent interferon response which is unable to control replication in lung epithelial cells. J. Virol. 95, e02415–e02420. doi: 10.1128/JVI.02415-20 PMC8103705 33514628

[B3] Ashrafzadeh-Kian S. Campbell M. R. Jara Aguirre J. C. Walsh J. Kumanovics A. Jenkinson G.. (2022). Role of immune mediators in predicting hospitalization of SARS-CoV-2 positive patients. Cytokine 150, 155790. doi: 10.1016/j.cyto.2021.155790 34991059 PMC8709828

[B4] Bojkova D. Rothenburger T. Ciesek S. Wass M. N. Michaelis M. Cinatl J. (2022). SARS-CoV-2 omicron variant virus isolates are highly sensitive to interferon treatment. Cell Discovery 8, 42. doi: 10.1038/s41421-022-00408-z 35538050 PMC9087166

[B5] Bruns A. M. Leser G. P. Lamb R. A. Horvath C. M. (2014). The innate immune sensor LGP2 activates antiviral signaling by regulating MDA5-RNA interaction and filament assembly. Mol. Cell 55, 771–781. doi: 10.1016/j.molcel.2014.07.003 25127512 PMC4156907

[B6] Chen T.-H. Hsu M.-T. Lee M.-Y. Chou C.-K. (2022). Gastrointestinal involvement in SARS-CoV-2 infection. Viruses 14, 1188. doi: 10.3390/v14061188 PMC9228950 35746659

[B7] Chow K. T. Gale M. Loo Y.-M. (2018). RIG-I and other RNA sensors in antiviral immunity. Annu. Rev. Immunol. 36, 667–694. doi: 10.1146/annurev-immunol-042617-053309 29677479

[B8] Divij M. Josephine R. G. Amy E. B. Derek A. O. Allison R. G. Jennifer E. W.. (2020). Deep immune profiling of COVID-19 patients reveals distinct immunotypes with therapeutic implications. Science 369, eabc8511. doi: 10.1126/science.abc8511 32669297 PMC7402624

[B9] Dusseaux M. Martin E. Serriari N. Péguillet I. Premel V. Louis D.. (2011). Human MAIT cells are xenobiotic-resistant, tissue-targeted, CD161hi IL-17–secreting T cells. Blood 117, 1250–1259. doi: 10.1182/blood-2010-08-303339 21084709

[B10] Elinav E. Strowig T. Kau A. L. Henao-Mejia J. Thaiss C. A. Booth C. J.. (2011). NLRP6 inflammasome regulates colonic microbial ecology and risk for colitis. Cell 145, 745–757. doi: 10.1016/j.cell.2011.04.022 21565393 PMC3140910

[B11] Fergusson J. R. Hühn M. H. Swadling L. Walker L. J. Kurioka A. Llibre A.. (2016). CD161intCD8+ T cells: A novel population of highly functional, memory CD8+ T cells enriched within the gut. Mucosal Immunol. 9, 401–413. doi: 10.1038/mi.2015.69 26220166 PMC4732939

[B12] Flament H. Rouland M. Beaudoin L. Toubal A. Bertrand L. Lebourgeois S.. (2021). Outcome of SARS-CoV-2 infection is linked to MAIT cell activation and cytotoxicity. Nat. Immunol. 22, 322–335. doi: 10.1038/s41590-021-00870-z 33531712

[B13] Guo M. Tao W. Flavell R. A. Zhu S. (2021). Potential intestinal infection and faecal–oral transmission of SARS-CoV-2. Nat. Rev. Gastroenterol. Hepatol. 18, 269–283. doi: 10.1038/s41575-021-00416-6 33589829 PMC7883337

[B14] Hage A. Bharaj P. van Tol S. Giraldo M. I. Gonzalez-Orozco M. Valerdi K. M.. (2022). The RNA helicase DHX16 recognizes specific viral RNA to trigger RIG-i-dependent innate antiviral immunity. Cell Rep. 38, 110434. doi: 10.1016/j.celrep.2022.110434 PMC8903195 35263596

[B15] Hoffmann M. Kleine-Weber H. Schroeder S. Krüger N. Herrler T. Erichsen S.. (2020). SARS-CoV-2 cell entry depends on ACE2 and TMPRSS2 and is blocked by a clinically proven protease inhibitor. Cell 181, 271–280.e8. doi: 10.1016/j.cell.2020.02.052 32142651 PMC7102627

[B16] Hur S. (2019). Double-stranded RNA sensors and modulators in innate immunity. Annu. Rev. Immunol. 37, 349–375. doi: 10.1146/annurev-immunol-042718-041356 30673536 PMC7136661

[B17] Jiang X. Kinch L. N. Brautigam C. A. Chen X. Du F. Grishin N.. (2012). Ubiquitin-induced oligomerization of the RNA sensors RIG-I and MDA5 activates antiviral innate immune response. Immunity 36, 959–973. doi: 10.1016/j.immuni.2012.03.022 22705106 PMC3412146

[B18] Jiao L. Li H. Xu J. Yang M. Ma C. Li J.. (2021). The gastrointestinal tract is an alternative route for SARS-CoV-2 infection in a nonhuman primate model. Gastroenterology 160, 1647–1661. doi: 10.1053/j.gastro.2020.12.001 33307034 PMC7725054

[B19] Jin X. Lian J.-S. Hu J.-H. Gao J. Zheng L. Zhang Y.-M.. (2020). Epidemiological, clinical and virological characteristics of 74 cases of coronavirus-infected disease 2019 (COVID-19) with gastrointestinal symptoms. Gut 69, 1002–1009. doi: 10.1136/gutjnl-2020-320926 32213556 PMC7133387

[B20] Ju X. Zhu Y. Wang Y. Li J. Zhang J. Gong M.. (2021). A novel cell culture system modeling the SARS-CoV-2 life cycle. PloS Pathog. 17, e1009439. doi: 10.1371/journal.ppat.1009439 33711082 PMC7990224

[B21] Karki R. Sharma B. R. Tuladhar S. Williams E. P. Zalduondo L. Samir P.. (2021). Synergism of TNF-α and IFN-γ triggers inflammatory cell death, tissue damage, and mortality in SARS-CoV-2 infection and cytokine shock syndromes. Cell 184, 149–168.e17. doi: 10.1016/j.cell.2020.11.025 33278357 PMC7674074

[B22] Kato H. Takeuchi O. Sato S. Yoneyama M. Yamamoto M. Matsui K.. (2006). Differential roles of MDA5 and RIG-I helicases in the recognition of RNA viruses. Nature 441, 101–105. doi: 10.1038/nature04734 16625202

[B23] Kell A. M. Gale M. (2015). RIG-I in RNA virus recognition. Virology 479–480, 110–121. doi: 10.1016/j.virol.2015.02.017 PMC4424084 25749629

[B24] Kenta M. Yusuke S. Seiko I.-K. Kazuo K. Tetsuo N. Atsushi M.. (2014). The DEAH-box RNA helicase DHX15 activates NF-κB and MAPK signaling downstream of MAVS during antiviral responses. Sci. Signal 7, ra40–ra40. doi: 10.1126/scisignal.2004841 24782566

[B25] Laing A. G. Lorenc A. del Molino del Barrio I. Das A. Fish M. Monin L.. (2020). A dynamic COVID-19 immune signature includes associations with poor prognosis. Nat. Med. 26, 1623–1635. doi: 10.1038/s41591-020-1038-6 32807934

[B26] Lazear H. M. Nice T. J. Diamond M. S. (2015). Interferon-λ: Immune functions at barrier surfaces and beyond. Immunity 43, 15–28. doi: 10.1016/j.immuni.2015.07.001 26200010 PMC4527169

[B27] le Bert N. Tan A. T. Kunasegaran K. Tham C. Y. L. Hafezi M. Chia A.. (2020). SARS-CoV-2-specific T cell immunity in cases of COVID-19 and SARS, and uninfected controls. Nature 584, 457–462. doi: 10.1038/s41586-020-2550-z 32668444

[B28] Leticia K.-C. Betina P. M. Wenzhao M. Aaron M. R. Caroline A. G. I. Ariel R. W.. (2020). Comprehensive mapping of immune perturbations associated with severe COVID-19. Sci. Immunol. 5, eabd7114. doi: 10.1126/sciimmunol.abd7114 32669287 PMC7402634

[B29] Levy M. Thaiss C. A. Zeevi D. Dohnalová L. Zilberman-Schapira G. Mahdi J. A.. (2015). Microbiota-modulated metabolites shape the intestinal microenvironment by regulating NLRP6 inflammasome signaling. Cell 163, 1428–1443. doi: 10.1016/j.cell.2015.10.048 26638072 PMC5665753

[B30] Livanos A. E. Jha D. Cossarini F. Gonzalez-Reiche A. S. Tokuyama M. Aydillo T.. (2021). Intestinal host response to SARS-CoV-2 infection and COVID-19 outcomes in patients with gastrointestinal symptoms. Gastroenterology 160, 2435–2450.e34. doi: 10.1053/j.gastro.2021.02.056 33676971 PMC7931673

[B31] Loo Y.-M. Gale J. M. (2011). Immune signaling by RIG-i-like receptors. Immunity 34, 680–692. doi: 10.1016/j.immuni.2011.05.003 21616437 PMC3177755

[B32] Lu Q. Liu J. Zhao S. Gomez Castro M. F. Laurent-Rolle M. Dong J.. (2021). SARS-CoV-2 exacerbates proinflammatory responses in myeloid cells through c-type lectin receptors and tweety family member 2. Immunity 54, 1304–1319.e9. doi: 10.1016/j.immuni.2021.05.006 34048708 PMC8106883

[B33] Lu H. Lu N. Weng L. Yuan B. Liu Y. Zhang Z. (2014). DHX15 senses double-stranded RNA in myeloid dendritic cells. J. Immunol. 193, 1364. doi: 10.4049/jimmunol.1303322 24990078 PMC4108507

[B34] Nie J. Li Q. Wu J. Zhao C. Hao H. Liu H.. (2020). Establishment and validation of a pseudovirus neutralization assay for SARS-CoV-2. Emerg. Microbes Infect. 9, 680–686. doi: 10.1080/22221751.2020.1743767 32207377 PMC7144318

[B35] Ou X. Liu Y. Lei X. Li P. Mi D. Ren L.. (2020). Characterization of spike glycoprotein of SARS-CoV-2 on virus entry and its immune cross-reactivity with SARS-CoV. Nat. Commun. 11, 1620. doi: 10.1038/s41467-020-15562-9 32221306 PMC7100515

[B36] Pan P. Shen M. Yu Z. Ge W. Chen K. Tian M.. (2021). SARS-CoV-2 n protein promotes NLRP3 inflammasome activation to induce hyperinflammation. Nat. Commun. 12, 4664. doi: 10.1038/s41467-021-25015-6 34341353 PMC8329225

[B37] Penghua W. Shu Z. Long Y. Shuang C. Wen P. Ruaidhri J.. (2015). Nlrp6 regulates intestinal antiviral innate immunity. Sci. (1979) 350, 826–830. doi: 10.1126/science.aab3145 PMC4927078 26494172

[B38] Polidoro R. B. Hagan R. S. de Santis Santiago R. Schmidt N. W. (2020). Overview: Systemic inflammatory response derived from lung injury caused by SARS-CoV-2 infection explains severe outcomes in COVID-19. Front. Immunol. 11. doi: 10.3389/fimmu.2020.01626 PMC7344249 32714336

[B39] Ricardo-Lax I. Luna J. M. Thi Nhu Thao T. le Pen J. Yu Y. Hoffmann H.-H.. Replication and single-cycle delivery of SARS-CoV-2 replicons. Available at: https://www.science.org. 10.1126/science.abj8430 PMC9007107 34648371

[B40] Rodrigues T. S. de Sá K. S. G. Ishimoto A. Y. Becerra A. Oliveira S. Almeida L.. (2020a). Inflammasomes are activated in response to SARS-CoV-2 infection and are associated with COVID-19 severity in patients. J. Exp. Med. 218, e20201707. doi: 10.1084/jem.20201707 PMC7684031 33231615

[B41] Shang J. Wan Y. Luo C. Ye G. Geng Q. Auerbach A.. (2020) Cell entry mechanisms of SARS-CoV-2. Proceedings of the National Academy of Sciences 117, 11727–11734 doi: 10.1073/pnas.2003138117/-/DCSupplemental PMC7260975 32376634

[B42] Shen C. Li R. Negro R. Cheng J. Vora S. M. Fu T.-M.. (2021). Phase separation drives RNA virus-induced activation of the NLRP6 inflammasome. Cell 184, 5759–5774.e20. doi: 10.1016/j.cell.2021.09.032 34678144 PMC8643277

[B43] Sherwani S. Khan M. W. A. (2020). Cytokine response in SARS-CoV-2 infection in the elderly. J. Inflammation Res. 13, 737–747. doi: 10.2147/JIR.S276091 PMC7585778 33116752

[B44] Stanifer M. L. Kee C. Cortese M. Zumaran C. M. Triana S. Mukenhirn M.. (2020). Critical role of type III interferon in controlling SARS-CoV-2 infection in human intestinal epithelial cells. Cell Rep. 32, 107863. doi: 10.1016/j.celrep.2020.107863 PMC7303637 32610043

[B45] Takeshi S. Reiko H. Yueh-Ming L. David O. Cynthia L. J. Sangita C. S.. (2007). Regulation of innate antiviral defenses through a shared repressor domain in RIG-I and LGP2. Proc. Natl. Acad. Sci. 104, 582–587. doi: 10.1073/pnas.0606699104 17190814 PMC1766428

[B46] Tao W. Zhang G. Wang X. Guo M. Zeng W. Xu Z.. (2020a). Analysis of the intestinal microbiota in COVID-19 patients and its correlation with the inflammatory factor IL-18. Med. Microecology 5, 100023. doi: 10.1016/j.medmic.2020.100023 PMC7832617 34173452

[B47] Tao W. Zhang G. Wang X. Guo M. Zeng W. Xu Z.. (2020b). Analysis of the intestinal microbiota in COVID-19 patients and its correlation with the inflammatory factor IL-18 and SARS-CoV-2-specific IgA. medRxiv 2020, 8.12.20173781. doi: 10.1101/2020.08.12.20173781 PMC7832617 34173452

[B48] van Dorp L. Acman M. Richard D. Shaw L. P. Ford C. E. Ormond L.. (2020). Emergence of genomic diversity and recurrent mutations in SARS-CoV-2. Infection Genet. Evol. 83, 104351. doi: 10.1016/j.meegid.2020.104351 PMC7199730 32387564

[B49] Verdonck S. Nemegeer J. Vandenabeele P. Maelfait J. (2022). Viral manipulation of host cell necroptosis and pyroptosis. Trends Microbiol. 30, 593–605. doi: 10.1016/j.tim.2021.11.011 34933805

[B50] Wang K. Chen W. Zhang Z. Deng Y. Lian J. Q. Du P.. (2020). CD147-spike protein is a novel route for SARS-CoV-2 infection to host cells. Signal Transduct Target Ther. 5, 283. doi: 10.1038/s41392-020-00426-x PMC7714896 33277466

[B51] WHO (2022). dashboard. WHO coronavirus disease (COVID-19). Available at: https://covid19.who.int/.

[B52] Wu H.-J. Wu E. (2012). The role of gut microbiota in immune homeostasis and autoimmunity. Gut Microbes 3, 4–14. doi: 10.4161/gmic.19320 22356853 PMC3337124

[B53] Wu F. Zhao S. Yu B. Chen Y.-M. Wang W. Song Z.-G.. (2020). A new coronavirus associated with human respiratory disease in China. Nature 579, 265–269. doi: 10.1038/s41586-020-2008-3 32015508 PMC7094943

[B54] Xiao F. Tang M. Zheng X. Liu Y. Li X. Shan H. (2020). Evidence for gastrointestinal infection of SARS-CoV-2. Gastroenterology 158, 1831–1833.e3. doi: 10.1053/j.gastro.2020.02.055 32142773 PMC7130181

[B55] Xie X. Muruato A. Lokugamage K. G. Narayanan K. Zhang X. Zou J.. (2020). An infectious cDNA clone of SARS-CoV-2. Cell Host Microbe 27, 841–848.e3. doi: 10.1016/j.chom.2020.04.004 32289263 PMC7153529

[B56] Xing J. Zhou X. Fang M. Zhang E. Minze L. J. Zhang Z. (2021). DHX15 is required to control RNA virus-induced intestinal inflammation. Cell Rep. 35, 109205. doi: 10.1016/j.celrep.2021.109205 PMC8276442 34161762

[B57] Yin X. Riva L. Pu Y. Martin-Sancho L. Kanamune J. Yamamoto Y.. (2021). MDA5 governs the innate immune response to SARS-CoV-2 in lung epithelial cells. Cell Rep. 34, 108628. doi: 10.1016/j.celrep.2020.108628 PMC7832566 33440148

[B58] Zang R. Florencia Gomez Castro M. Mccune B. T. Zeng Q. Rothlauf P. W. Sonnek N. M.. (2020) TMPRSS2 and TMPRSS4 promote SARS-CoV-2 infection of human small intestinal enterocytes. Available at: https://www.science.org. 10.1126/sciimmunol.abc3582 PMC7285829 32404436

[B59] Zeng W. Sun L. Jiang X. Chen X. Hou F. Adhikari A.. (2010). Reconstitution of the RIG-I pathway reveals a signaling role of unanchored polyubiquitin chains in innate immunity. Cell 141, 315–330. doi: 10.1016/j.cell.2010.03.029 20403326 PMC2919214

[B60] Zhou J. Li C. Liu X. Chiu M. C. Zhao X. Wang D.. (2020). Infection of bat and human intestinal organoids by SARS-CoV-2. Nat. Med. 26, 1077–1083. doi: 10.1038/s41591-020-0912-6 32405028

