# Supplementary material for: SARS-CoV-2 infection of intestinal epithelia cells sensed by RIG-I and DHX-15 evokes innate immune response and immune cross-talk
Source: Front Cell Infect Microbiol. 2023 Feb 7;12:1035711. doi: 10.3389/fcimb.2022.1035711 (PMC9941539; doi:10.3389/fcimb.2022.1035711)
Supplement: Supplementary file 1 [file DataSheet_1.docx]

**Supplemental Materials**

**SARS-CoV-2 infection of intestinal epithelia cells sensed by RIG-I and DHX-15 evokes innate immune response and immune cross-talk**

Lijuan Zhang^1,6^, Yize Zhang^2,6^, Ruiqin Wang^3^, Xiaoning Liu^1^, Jinmeng Zhao^4^, Masato Tsuda^5^, You Li*^1,3^

^1^School of Medicine, [Huanghe Science and Technology College](https://www.4icu.org/reviews/802.htm), Zhengzhou, China.

^2^Precision Medicine Center, Gene Hospital of Henan Province, The First Affiliated Hospital of Zhengzhou University, Zhengzhou, China.

^3^School of Life Science and Technology, Tongji University, Shanghai, China.

^4^School of Life Science, Zhengzhou University, Zhengzhou, China.

^5^School of Medicine, Niigata University, Niigata, Japan.

^6^These authors contributed equally to the work.


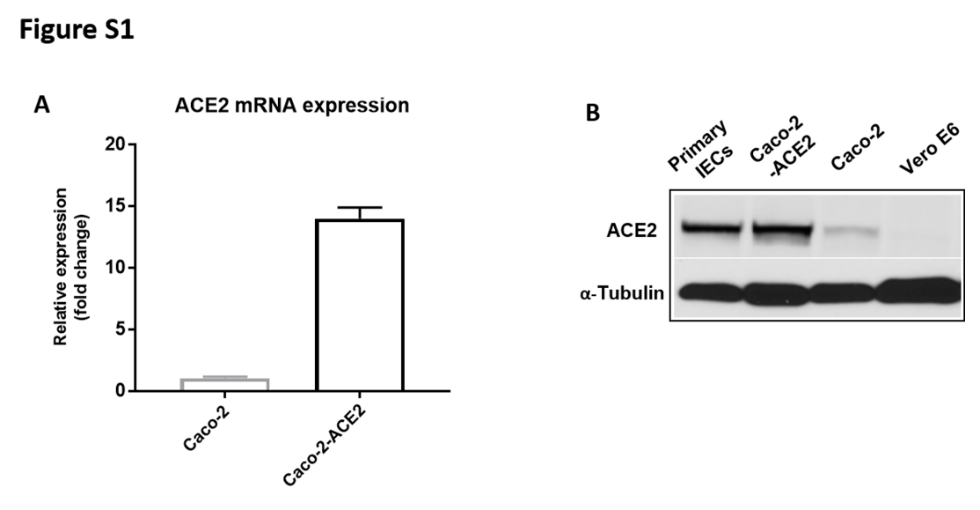


**Figure S1. Overexpression of ACE2 in Caco-2 cell by stable transduction.** (A) mRNA level of ACE2 in Caco-2 and ACE2 overexpressed Caco-2-ACE2 cells; (B) Western blot of ACE2 expression in primary epithelial cells and cell lines.


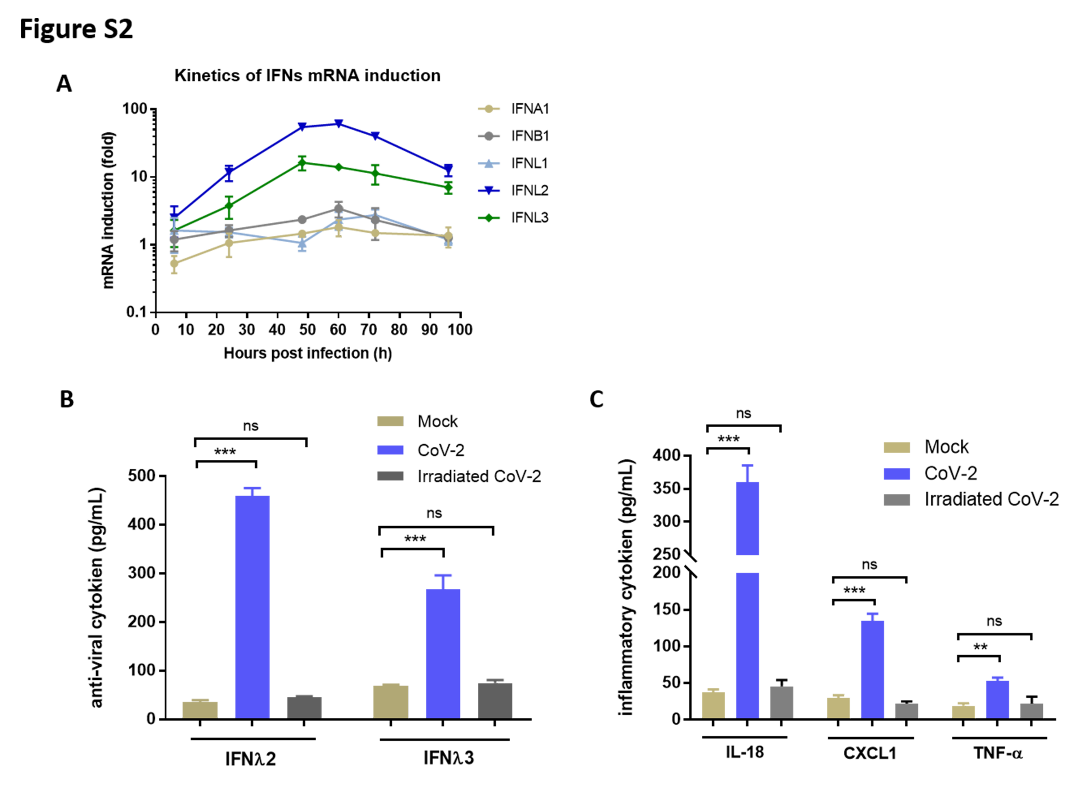


**Figure S2. Anti-viral and inflammatory cytokines induction in Caco-2-ACE2-N cells response to SARS-CoV-2 GFP/ΔN trVLP infection.** Caco-2-ACE2-N cells (2x10^5^) were infected with mock or SARS-CoV-2 GFP/ΔN trVLP (CoV-2) with MOI = 0.25. (A) The kinetics of IFNs mRNA induction over the course of SARS-CoV-2 infection in Caco-2-ACE2-N cells. (B) At 48h post infection, supernatant was harvested and anti-viral cytokines IFNλ2 and IFNλ3 were detected by ELISA. (C) At 72h post infection, inflammatory cytokines IL-18, CXCL1, TNF-α were determined by ELISA respectively. Data are represented as mean ± SEM of four replicates. Statistical significance is indicated. (*p<0.05, **p<0.01 ***p<0.001, One-way ANOVA.)


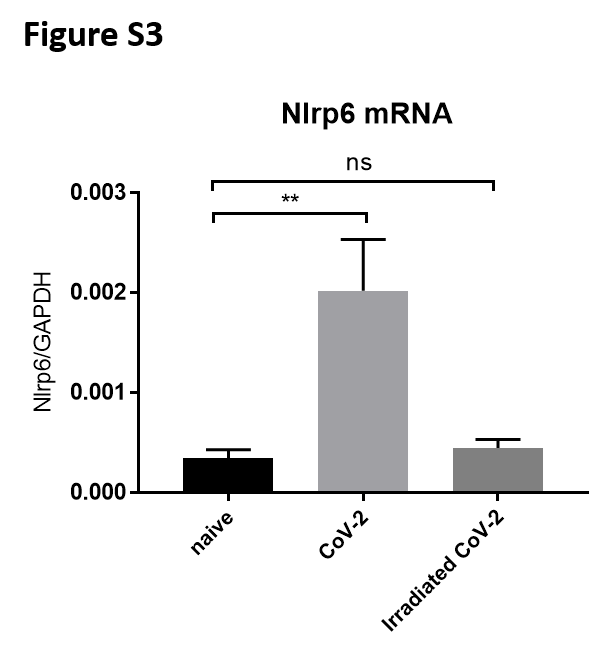


**Figure S3. Competent CoV-2 infection but not irradiated CoV-2 itself increased Nlrp6 expression in Caco-2-ACE2-N cells upon SARS-CoV-2 GFP/ΔN trVLP infection.** Caco-2-ACE2-N cells were infected with CoV-2 or irradiated CoV-2 at 0.5 MOI. At 16 hpi, mRNA level of nlrp6 was quantified by qRT-PCR.
